# Harnessing the power of microbial nanowires

**DOI:** 10.1111/1751-7915.13280

**Published:** 2018-05-27

**Authors:** Gemma Reguera

**Affiliations:** ^1^ Department of Microbiology and Molecular Genetics Michigan State University 567 Wilson Rd., Rm. 6190 East Lansing MI 48824 USA

## Abstract

The reduction of iron oxide minerals and uranium in model metal reducers in the genus *Geobacter* is mediated by conductive pili composed primarily of a structurally divergent pilin peptide that is otherwise recognized, processed and assembled in the inner membrane by a conserved Type IVa pilus apparatus. Electronic coupling among the peptides is promoted upon assembly, allowing the discharge of respiratory electrons at rates that greatly exceed the rates of cellular respiration. Harnessing the unique properties of these conductive appendages and their peptide building blocks in metal bioremediation will require understanding of how the pilins assemble to form a protein nanowire with specialized sites for metal immobilization. Also important are insights into how cells assemble the pili to make an electroactive matrix and grow on electrodes as biofilms that harvest electrical currents from the oxidation of waste organic substrates. Genetic engineering shows promise to modulate the properties of the peptide building blocks, protein nanowires and current‐harvesting biofilms for various applications. This minireview discusses what is known about the pilus material properties and reactions they catalyse and how this information can be harnessed in nanotechnology, bioremediation and bioenergy applications.

## Introduction

The evolution of biological complexity is intimately connected with molecular self‐assembly processes that spontaneously arrange proteins or peptides in precise configurations to enable new functions. The geometric assembly of proteins leads, for example, to the formation of molecular nanomachines and hyperstructures such as the ATP synthase complex and the cytoskeletal microtubules respectively (Alfaro‐Aco and Petry, 2015; Ruhle and Leister, 2015). Proteins can also self‐assemble in planar geometric configurations to make the S‐layer lattices of some bacteria and archaea (Sleytr *et al*., 2014) and into distinctive 3D geometries such as bacterial intracellular microcompartments and viral capsids (Uetrecht *et al*., 2011; Sutter *et al*., 2017). These natural designs have inspired the synthesis of novel nanomaterials and protocols for protein functionalization and controlled association that modulate the material's properties and enable new functions (Yang *et al*., 2016). The self‐assembly properties of some peptides have also attracted substantial interest in nanotechnology. The osteointegration of orthopaedic and dental titanium implants can, for example, be improved with coatings of bifunctional peptides selected for their ability to simultaneously bind the titanium alloy and the targeted tissue (Yazici *et al*., 2013). Peptide self‐assemblies have also inspired designs of molecular surface coatings used in cell adhesion assays (Chen *et al*., 1997) and biosensors (Templin *et al*., 2002; Bertone and Snyder, 2005; Ma *et al*., 2012; Gupta *et al*., 2016). Moreover, peptides can be printed onto solid surfaces in defined array geometries to develop platforms for the screening of antibodies (immunoassays), other proteins and peptides (study of protein–protein interactions), synthetic ligands (drug discovery) or small molecules (protein–metabolite interactions) (Gupta *et al*., 2016).

Many bacteria rely on the self‐assembly of helical peptides known as pilins to form Type IV pili (T4P), protein appendages that protrude outside the cells to perform a variety of extracellular functions (Craig and Li, 2008; Maier and Wong, 2015). The ability of T4 pilins to polymerize spontaneously via hydrophobic interactions has inspired ‘bottom‐up’ fabrication protocols for the synthesis of protein nanotubes (Audette *et al*., 2004a,b) and biocoatings that prevent the corrosion of metallic implants (Muruve *et al*., 2016). The discovery that metal‐reducing bacteria in the genus *Geobacter* produce conductive T4P for the reduction of ferric (Fe[III]) oxide minerals (Reguera *et al*., 2005) and the uranyl cation (Cologgi *et al*., 2011) suggests novel applications are to be harnessed from the unique electronic and metal binding properties of these protein filaments and their peptide building blocks.

The ability of *Geobacter* T4P to function as protein nanowires contrasts with other metal‐reducing bacteria such as *Shewanella* species, which secrete soluble flavin mediators to shuttle respiratory electrons to extracellular electron acceptors (Kotloski and Gralnick, 2013). The model representative *Shewanella oneidensis* MR‐1 was also assumed to produce pilus ‘nanowires’ (Gorby *et al*., 2006; El‐Naggar *et al*., 2010), although MR‐1 T4P had previously been shown to be nonconductive (Reguera *et al*., 2005). These ‘nanowires’ were later found to be dehydrated forms of outer membrane extensions, which this bacterium forms by fusing outer membrane vesicles (Pirbadian *et al*., 2014). Theoretical studies suggest that the clusters of outer membrane *c‐*type cytochromes (c‐Cyts) that decorate the MR‐1 extensions could diffuse and collide to enable charge transport *in vivo* (Subramanian *et al*., 2018). However, this model is yet to be validated experimentally. Thus, the T4P of *Geobacter* bacteria are to this date the only microbial protein appendages with demonstrated capacity to function as nanowires.

**Figure 1:**
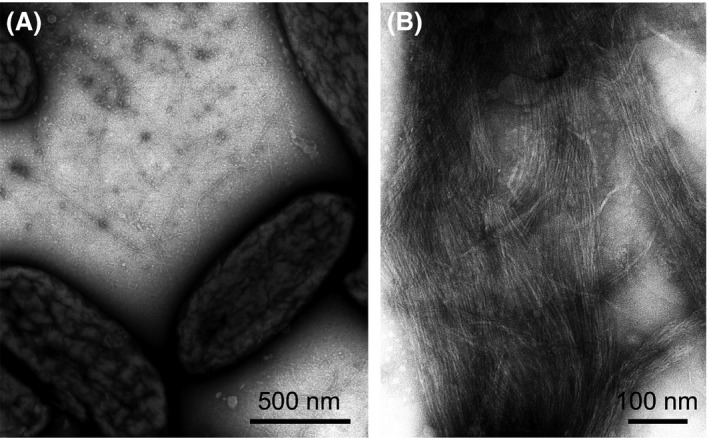
Transmission Electron Micrograph (TEM) of negatively stained piliated cells of *Geobacter sulfurreducens* (A) and purified pili (B).

The conductive T4P of *Geobacter* also mediate cell–cell aggregation (Fig. 1) and the formation of biofilms on electrodes that harvest electrical currents from the oxidation of organic acids (Reguera *et al*., 2006, 2007; Steidl *et al*., 2016). Genetic engineering can be applied to improve the robustness of the electrode‐associated biofilms at the oxidation of mixes of fermentation byproducts under industrial relevant conditions (Speers and Reguera, 2012a,b; Speers *et al*., 2014). Furthermore, the bioelectrodes can be retrofitted into fermenters to prevent the accumulation of unwanted products, simultaneously improving the efficiency of the fermentation and enriching for the added‐value chemical to facilitate its purification in downstream steps (Awate *et al*., 2017). By powering electro‐fermentations, the electroactive biofilms improve the economic feasibility of fermentation‐based approaches that harvest energy from waste organic substrates (Awate *et al*., 2017).

Harnessing the biological reactions catalysed by the conductive T4P will require better understanding of the material's properties and the biological mechanisms that allow *Geobacter* cells to integrate these protein nanowires in the respiratory machinery of the cell. This mini‐review highlights the seminal studies that have provided novel insights into these critical aspects of T4P functionality, highlighting those that the author views as having the most significant potential to harness the unique properties of these protein nanowires in energy‐related applications, nanotechnology and bioremediation.

## A nanowire pathway for metal reduction

T4P are produced by a wide variety of Gram‐negative bacteria, from pathogens such as *Pseudomonas* and *Neisseria* spp. to environmental bacteria in the *Myxococcus* and *Shewanella* genera, and they have more recently been described in Gram‐positive bacteria (Pelicic, 2008). They are dynamic filaments that undergo cycles of polymerization and depolymerization of primarily one pilin peptide, to promote, among other functions, attachment to and/or translocation on surfaces, binding and uptake of DNA, and/or cell–cell aggregation and biofilm formation (Craig and Li, 2008; Maier and Wong, 2015). The genomes of several *Geobacter* bacteria encode all of the genes needed to make a functional T4P apparatus, but the pilin gene encodes a uniquely short peptide (Reguera *et al*., 2005). Genetic studies in the model representative *Geobacter sulfurreducens* (GS) have provided insights into how the cells assemble the GS pilin into a conductive fibre. However, little is known about how respiratory electrons are discharged through the T4P, partly because mutations that inactivate key electron carriers of the cell envelope, whether the T4P or c‐Cyts, are often pleiotropic (Kim *et al*., 2005, 2006; Kim and Lovley, 2008; Cologgi *et al*., 2011; Richter *et al*., 2012; Steidl *et al*., 2016). As a result, compensatory effects are often observed that can mask the true phenotype of the mutants. These studies highlight how tightly integrated are the T4P in the respiratory machinery of the cell envelope, where c‐Cyts are the most abundant electron carriers. A few inactivating T4P mutations have been reported, however, which do not affect the expression of c‐Cyts (Richter *et al*., 2012; Speers *et al*., 2016; Steidl *et al*., 2016). These studies are particularly important to understand the role of the conductive T4P as electronic conduits between the cell and extracellular electron acceptors.

### Conductive T4P and c‐Cyts team up

**Figure 2:**
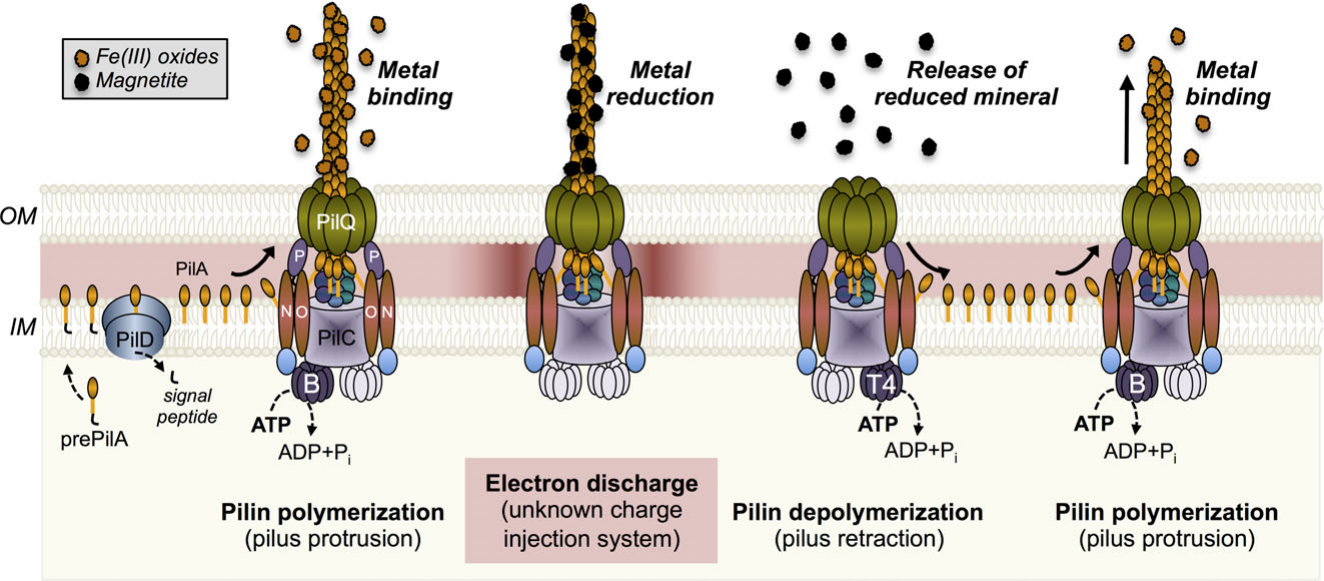
Components of the GS T4P and their role in the dynamic protrusion and retraction of the pilus fibre during the reduction of Fe(III) oxides to magnetite (grey legend) and soluble Fe(II) (not shown). The textboxes at the bottom of the illustration indicate the sequential steps of prepilin (prePilA) processing by the PilD signal peptidase in the inner membrane (IM), the polymerization of the mature pilin (PilA) and pilus protrusion across the outer membrane (OM), electron discharge, and pilus depolymerization to recycle the pilins in the inner membrane for a new round of polymerization. The corresponding extracellular steps of metal binding, reduction, and release of the reduced mineral are shown on top (italicized and in bold).

*Geobacter* T4P are assembled on one side of the cell only (Reguera *et al*., 2005) (Fig. 1). This contrasts with the preferential polar localization of T4P in other bacteria, which promotes the formation of a polar, retractable bundle of T4P to reduce cell surface friction and enhance the pulling motion needed to translocate cells on surfaces (Mattick, 2002; Jarrell and McBride, 2008). Perhaps not surprisingly, T4P‐mediated motility has never been demonstrated in *Geobacter* bacteria although cells have functional T4P motors needed to retract the fibres (Speers *et al*., 2016). Yet, the monolateral assembly of conductive T4P in *Geobacter* concentrates a great number of the appendages on one side of the cell, expanding its redox‐active surface beyond the confines of the outer membrane and maximizing the chances of establishing electronic contact with the extracellular electron acceptor. This is particularly important during the reduction in Fe(III) oxides, the natural electron acceptor for *Geobacter* bacteria (Fig. 2). Fe(III) oxide minerals are particularly abundant in sediments and soils (Straub *et al*., 2005). The most bioavailable of the Fe(III) oxide phases (e.g. ferrihydrate) is dispersed in these environments and transform rapidly into more crystalline mineral phases such as goethite and haematite (Lentini *et al*., 2012). The dense monolateral network of conductive T4P allows the cell to rapidly access the dispersed minerals so they can be reduced before they are abiotically transformed into more recalcitrant phases. Discharging electrons onto the minerals is predicted to be fast. The charge transport capacity of individual pilus fibres purified free of metal and organic cofactors (~ 1 billion electrons per second at 100 mV) is, for example, two orders of magnitude greater than the rates of iron oxide respiration per cell (Lampa‐Pastirk *et al*., 2016). These measurements were obtained for purified pilus fibres deposited onto a substrate without extensive evaporation and/or chemical fixation (Lampa‐Pastirk *et al*., 2016). Although these conditions preserved the structural and electronic signatures described for cell‐associated pili (Veazey *et al*., 2011), the assay was performed with T4P immobilized on a surface. This contrasts with the *in‐vivo* conditions, where the fibres undergo antagonistic cycles of protrusion and retraction and experience motions that are predicted to promote electronic coupling and charge transport (Feliciano *et al*., 2015). Thus, the charge transport rates estimated *in vitro* (Lampa‐Pastirk *et al*., 2016) may in fact underestimate the true transport capacity of the T4P *in vivo*. Furthermore, numerous fibres protrude from one side of the cell at any given time (Fig. 1), providing many opportunities for electronic contact with the extracellular electron acceptor and rapid discharges of respiratory electrons.

The conductive T4P are also the primary mechanism for the reduction of the uranyl cation (UO_2_^2+^) (Cologgi *et al*., 2011). The fibres bind the soluble cation extracellularly and discharge respiratory electrons to reduce the U(VI) to a mononuclear U(IV) mineral phase (Cologgi *et al*., 2011). This reductive reaction effectively mineralizes the radionuclide outside the cell and prevents its permeation and nonspecific reduction in the cell envelope (Cologgi *et al*., 2011). Thus, the protein nanowires provide a dual strategy for energy conservation and cellular protection from the toxic uranyl cation. *Geobacter* cells also express substantial quantities of c‐Cyts during the reduction in metals (Shi *et al*., 2016). These c‐Cyts localize to the inner membrane, periplasmic space and outer membrane, providing a potential path for the transfer of electrons to the cell surface. Micrographs of uranium‐reducing cells show some spots of uranium mineralization on the cell surface, which could correspond to reductive foci of outer membrane c‐Cyts (Cologgi *et al*., 2011). However, in contrast to the positive correlation between piliation and extracellular uranium mineralization (Cologgi *et al*., 2011), a reverse correlation exists between the outer membrane haem content and the amount of uranium that is mineralized outside the cell and prevented from traversing the outer membrane (Reguera, 2012). This suggests that outer membrane c‐Cyts provide secondary reductive foci on the outer membrane, yet their contribution to mineralization is small compared to the conductive T4P.

Although abundant in the cell envelope, there is little sequence conservation among c‐Cyts from *Geobacter* bacteria (Butler *et al*., 2010). This suggests that c‐Cyt abundance and perhaps the redox potentials that they operate at, rather than specific c‐Cyts, may be important during metal reduction. The periplasmic c‐Cyts PpcA and PpcD are particularly abundant in the cell envelope and are conserved among *Geobacter* species (Butler *et al*., 2010). These c‐Cyts are predicted to contribute to the proton motive force by coupling the transport of electrons to protons, although using different mechanisms (Pessanha *et al*., 2006; Morgado *et al*., 2010). Energy transduction by these c‐Cyts can be sustained over a wide range of redox potentials (mid‐point potential versus the standard hydrogen electrode ranges from −0.167 to −0.109 V in PpcA and from −0.202 to −0.146 V in PpcD), allowing them to function as intermediate electron carriers to more than one redox partner (Morgado *et al*., 2010). The exposure of the base of the T4P fibres in the periplasm offers opportunities for interactions with the periplasmic electron carriers and electron discharges via the T4P pathway (Fig. 2). Periplasmic c‐Cyts have also been proposed to contribute to the capacitance of the cell envelope. The content of c‐Cyts in the cell envelope increases substantially under electron acceptor limitation and promotes the storage of approximately 10^7^ electrons per cell (Esteve‐Nunez *et al*., 2008). This storage capacity is estimated to allow cells to support maintenance metabolic rates for 8 minutes (Esteve‐Nunez *et al*., 2008). By functioning as a capacitor, the abundant periplasmic c‐Cyts could store electrons at the base of the T4P until the appendages make electronic contact with the extracellular electron acceptor and the charges can be discharged (Fig. 2). Furthermore, the storage of electrons in the c‐Cyts maintains proton pumping across the inner membrane, sustaining the electrochemical gradient that supports ATP synthesis in the cytoplasm. This provides a mechanism to energize cycles of pilin polymerization and depolymerization to maximize the chances of establishing productive interactions between the T4P and the extracellular electron acceptor (Fig. 2). At this point, the c‐Cyt capacitors could function as a charge injection system, discharging the electrons via the T4P and reducing the T4P‐bound electron acceptor (Fig. 2).

### Dynamic assembly of the GS pilin during metal reduction

The *Geobacter* T4P pili consist primarily of a single pilin peptide, or PilA (Cologgi *et al*., 2011). The peptide is synthesized as a precursor (prepilin) carrying a leader peptide (Richter *et al*., 2012). The sequence and length of this leader peptide have been traditionally used to classify bacterial pilins in the T4a and T4b classes (Giltner *et al*., 2012). At the sequence level, the GS leader peptide has the conserved features of T4a pilins, including a conserved recognition site for its cleavage by a dedicated, membrane‐bound peptidase (PilD) (Richter *et al*., 2012) (Fig. 2). Furthermore, upon cleavage, a mature peptide is produced with a conserved phenylalanine amino acid in position +1 (E1), which is *N*‐methylated in most T4a pilins prior to the assembly of the peptide (Giltner *et al*., 2012). The leader peptides of T4a pilins also tend to be shorter (6–7 residues long) than those carried by T4b pilins (15 to 30 residues) (Giltner *et al*., 2012). However, transcriptional and biochemical studies (Juarez *et al*., 2009; Richter *et al*., 2012) suggest that the GS prepilin is synthesized as two isoforms carrying a short (10 amino acids) and long (29 amino acids) leader peptide. Both isoforms are recognized and cleaved at the inner membrane by the PilD peptidase. Yet, the short isoform accumulates in the cytoplasm and plays roles in the cytoplasmic stabilization of the long isoform and the secretion of some outer membrane c‐Cyts (Richter *et al*., 2012). Post‐translational modification of the pilin's tyrosine 32 with a glycerophosphate has also been proposed to be important for pili functionality (Richter *et al*., 2017).

The assembly of the mature GS pilin is predicted to follow the conserved mechanisms of other bacterial T4P (Fig. 2). The proteins encoded in the *pilMNOPQ* cluster initiate the formation of the biogenesis apparatus across the outer and inner membranes (Karuppiah *et al*., 2013). In *Myxococcus xanthus,* another delta‐proteobacterium, assembly is initiated with the formation of the PilQ secretin ring on the outer membrane and the recruitment of the inner membrane PilP lipoprotein and the PilNO subcomplex (Friedrich *et al*., 2014). The cytoplasmic protein PilM is then recruited by PilNO, an association that changes the orientation of PilM to promote interactions with the assembly protein PilC at the base of the pilus (Friedrich *et al*., 2014). As in *M. xanthus*, the GS PilC is encoded in a separate gene cluster (*pilBTCSRA)* containing the genes that code for the sensor (*pilS*) and response regulator (*pilR*) of pilus biogenesis (Juarez *et al*., 2009) and the operon containing the *pilA* gene (*pilA‐N*, encoding the PilA pilin) and a gene encoding a hypothetical protein (*pilA‐C*) (Reguera *et al*., 2005). The cluster also includes the *pilB* and *PilT4* genes, which code for the PilB and PilT4 ATPases that interact with PilC to power the extension and retraction of the T4P respectively (Speers *et al*., 2016; Steidl *et al*., 2016). The antagonistic cycles of protrusion and retraction powered by PilB and PilT4 allow the conductive T4P to effectively bind and reduce extracellular metals and then detach the reduced minerals, respectively (Fig. 2). Furthermore, the dynamic cycles of polymerization and depolymerization recycle the pilins in the membrane allowing the cells to repolymerize the T4P rapidly and continue to breath (Speers *et al*., 2016).

The GS genome harbors three other PilT paralogues, but only PilT4 is essential for metal reduction (Speers *et al*., 2016). Consistent with its role as the main retraction ATPase, PilT4 rescues the T4P retraction and twitching motility defects of a PilT‐deficient mutant of *P. aeruginosa* (Speers *et al*., 2016). The GS PilT paralogue PilT3 also rescues twitching motility in the PilT‐deficient mutant of *P. aeruginosa* and is the only GS PilT paralogue that restores the piliation levels of planktonic cells and the biofilm thickness of the hyperpiliated *Pseudomonas* PilT^‐^ strain (Speers *et al*., 2016). Yet, the inactivation of GS PilT3 in its native host only causes delays in the initiation of Fe(III) oxide reduction and biofilm formation, in agreement with the reduced adhesion phenotypes reported for secondary PilT motors (Speers *et al*., 2016). This suggests that PilT3 works coordinately with the main PilT motor, PilT4, to modulate the retraction of the T4P during metal reduction and biofilm formation.

By fine‐tuning T4P retraction, PilT3 could modulate the frequency and force of pilin depolymerization, as reported for the PilT paralogues of *M. xanthus* (Clausen *et al*., 2009), but adapted to the unique role of the GS T4P in metal respiration. The reduction of Fe(III) oxides by the conductive T4P, for example, solubilizes one‐third of the ferric iron (Fe[III]) as the ferrous ion (Fe[II]_aq_) and leaves the remaining Fe(II) as magnetite, a mineral of mix ferric‐ferrous valence that remains bound to the T4P (Fig. 2) (Reguera *et al*., 2005). Yet, the reduction of the uranyl cation produces a fine‐grained, mononuclear mineral phase of U(IV) (Cologgi *et al*., 2011), whose effective detachment from the T4P may require less retraction forces. However, the frequency of retraction may increase during the respiration of the uranyl cation to prevent its permeation and mineralization inside the cell envelope. In support of this, the respiration of the uranyl cation by GS cells generates less energy for growth than predicted thermodynamically (Sanford *et al*., 2007). This could be due to the increased energy cost of powering more frequent cycles of T4P protrusion and retraction.

## *Geobacter* T4P: a paradigm in structure and function

### The GS pilin is a divergent T4a pilin

**Figure 3:**
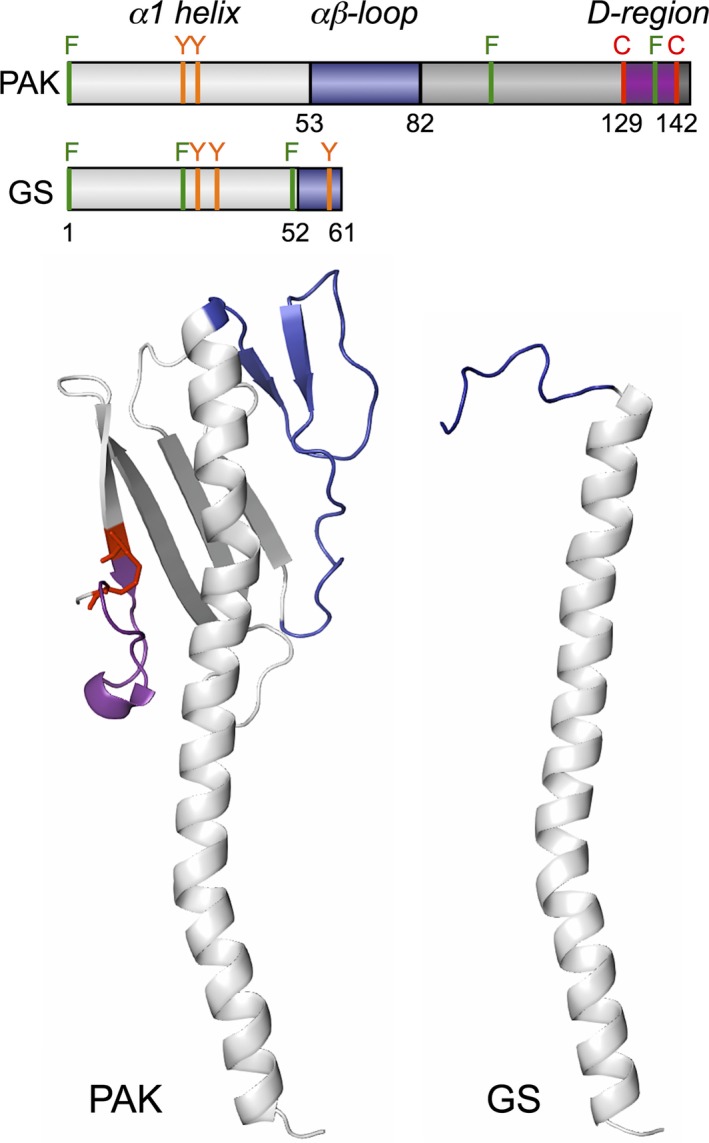
Domain architecture (top) and molecular structure (bottom) of the PAK (PDB, 1oqw) and the GS (PDB, 2m7g) pilins. The PAK has the canonical architecture of bacterial pilins: α1‐helix (light grey) and αβ‐loop (blue) domains followed by a β‐stranded region (dark grey) and the conserved D‐region (purple) containing a disulphide bond between two cysteine residues (in red). The structure of the GS pilin is truncated and reduced to an α1‐helix domain and a short random coiled segment at the carboxy‐terminus (in blue). The distribution of aromatic residues (F, phenylanine, in green; Y, tyrosine, in orange) along the PAK and GS pilin sequences is also shown in the top panel.

The most distinctive feature of the GS pilin is perhaps its short size (Reguera *et al*., 2005). This has major structural implications that are relevant to its role as a building block to make conductive T4P (Feliciano *et al*., 2012). Figure 3 illustrates this by comparing the GS pilin to the pilin of *P. aeruginosa* strain K (PAK pilin), which serves as a structural model for other T4a pilins (Hazes *et al*., 2000; Craig *et al*., 2003; Audette *et al*., 2004a,b; Winther‐Larsen *et al*., 2007). The mature GS pilin is, for example, 61 amino acids long, whereas T4a pilins are typically 150–175 amino acids long (Giltner *et al*., 2012). Despite being relative short (142‐amino acids long), the PAK pilin retains the domain architecture of bacterial T4a pilins, which comprises an α1 domain (an α‐helical, amino‐terminal domain spanning approximately 53 amino acids) and a carboxy‐terminal globular head with an αβ‐loop domain, an antiparallel β‐sheet region, and a carboxy‐terminal D‐region flanked by two conserved cysteines (Fig. 3). Electrostatic interactions between the β‐sheet region and amino acids of the top half of the helix domain (α1‐C helix) link the two structural modules together and allow the globular head to engulf as much as half of the upper portion of the peptide's α‐helix (Craig and Li, 2008). By contrast, the structure of the GS pilin resolved in micelles by NMR (Reardon and Mueller, 2013) is that of a peptide with the conserved hydrophobic α1 domain, which spans approximately 52 amino acids, and a short (9 amino acids) carboxy‐terminal random coiled segment (Fig. 3). The GS α1 domain also retains a phenylalanine in position +1 (F1) and a glutamic acid residue in position +5 (E5), which interact in neighbouring pilins to align the peptides during assembly (Feliciano *et al*., 2015; Steidl *et al*., 2016). Thus, the GS pilin retains the conserved structure (α1 domain) and amino acids (F1 and E5 residues) needed for pilin assembly (Feliciano *et al*., 2015). But it lacks the globular head that neutralizes the natural dipole of the α1 domain in other pilins. As a result, the GS pilin has a strong electrostatic field along the helix axis that is predicted to accelerate the rates of electron transfer through the helical peptide (Feliciano *et al*., 2012). Furthermore, the GS pilin lacks the more insulating β‐strand conformations (Shin *et al*., 2003), a structural property that contributes to creating a peptide environment optimal for charge transport (Feliciano *et al*., 2012). Consistent with these predictions, the GS pilin assembly is conductive whereas PAK pilins form insulating T4P (Lampa‐Pastirk *et al*., 2016).

Phylogenetic analyses of bacterial T4 pilins and their close relatives (pseudopilins) in Type II secretion systems place the GS pilin in an independent line of descent with other short T4a pilins in the order *Desulfuromonadales* (Reguera *et al*., 2005). As of 2016, the clade of short GS‐like pilins had been estimated to comprise 77% of all the T4a sequences in the *Desulfuromonadales* (Holmes *et al*., 2016). Sequence comparisons within the clade reveal a high degree of conservation, as expected of proteins (and genes) subjected to strong positive selection (Holmes *et al*., 2016). This is consistent with the specialized role of these T4a pilins in the formation of protein nanowires for the reduction in soluble and insoluble metals. Also, unlike other T4a pilins, the gene encoding the precursor of the short pilins (*pilA‐N*) is organized in an operon with a gene (*pilA‐C*) coding for a hypothetical protein (Reguera *et al*., 2005; Holmes *et al*., 2016). The PilA‐C protein contains domains that could fold as antiparallel β‐sheets, a structural feature found in the globular head of canonical T4a pilins (Shu *et al*., 2016). This has led to the suggestion that the genes encoding PilA‐N (pilin precursor) and PilA‐C (hypothetical protein) originated from the duplication of an ancestral full‐length pilin gene that lost the peptide's carboxyl‐ (*pilA‐N*) or amino‐ (*pilA‐C*) terminal regions (Shu *et al*., 2016). The need to respire extracellular electron acceptors may indeed have exerted evolutionary pressure on an ancestral full‐length pilin to lose the more insulating β‐strands and acquire a predominantly α‐helical configuration for increased electronic coupling (Feliciano *et al*., 2012). This event could explain the evolution of the short PilA‐N pilins but is unlikely to explain how it could have selected for the β‐stranded PilA‐C peptide. Furthermore, *pilA‐C* sequences, although under positive selection, are highly divergent and cluster in two separate phylogenetic clades, suggesting they evolved from different ancestral sequences (Holmes *et al*., 2016). This suggests that Pil‐C could play biological functions relevant to pili‐mediated functions in the environment, yet it is unlikely to share the same pilin ancestor.

### Coherent versus incoherent T4P conductance, that is the question

*Ab initio* simulations of the GS pilin modelled in solution reveal contributions of aromatic residues (tyrosines and phenylalanines) to the HOMO (Highest Occupied Molecular Orbital) and LUMO (Lowest Unoccupied Molecular Orbital) states that could promote intramolecular electron transfer reactions (Feliciano *et al*., 2012). The HOMO and LUMO regions are also located in regions of the pilin predicted to align once the peptides assemble, offering opportunities for intermolecular electron transfer via HOMO–LUMO and LUMO–LUMO interactions (Feliciano *et al*., 2012). Consistent with this, scanning tunnelling microscopy (STM) of the conductive GS T4P reveals a rich electronic molecular structure (Veazey *et al*., 2011) that is absent in the insulating PAK T4P (Lampa‐Pastirk *et al*., 2016). The electronic features of individual GS fibres resolved in STM images have periodicities matching the structural periodicities (major and minor grooves) of other bacterial T4P (Veazey *et al*., 2011), which result from the alignment of T4a pilins with a conserved 10.5‐Å rise (Craig *et al*., 2006). However, the periodic structural features of the GS T4P have electronic states near the Fermi level that are typical of conducting materials (Veazey *et al*., 2011).

The unique electronic structure of the GS T4P has motivated studies aimed at understanding the mechanism underlying its conductivity. One way to do this is to investigate how the conductivity of the material responds to extrinsic parameters such as temperature and pH. Crude preparations of pili sheared off the cell and dried overnight on interdigitated gold electrodes are conductive and display metallic‐like responses to temperature and pH (Malvankar *et al*., 2011). These results are, however, difficult to interpret because the redox‐active haem groups from c‐Cyts remain in the pili sample, even after treatment with a reductant to unfold the haem‐containing proteins. Furthermore, the native structure of the T4P could have been compromised after extensively drying the sample onto the electrodes prior to probing its conductivity (Strycharz‐Glaven and Tender, 2012). The crude sample was also analysed by X‐ray powder diffraction and the *d*‐spacing of the diffraction peak at 25^°^ (3.5 Å) was interpreted as an indication of π‐orbital overlap and charge delocalization caused by the stacking of aromatic side‐chains in π‐π configurations (Malvankar *et al*., 2011). Concerns have been raised about this interpretation as well, partly because the contribution from c‐Cyts and other sample contaminants to the diffraction effects was never assessed (Strycharz‐Glaven and Tender, 2012). Electrochemical studies of pili preparations spun coated onto interdigitated electrodes and probed in an aqueous buffered environment also suggest that the GS T4P could support ohmic electron conduction in solution (Ing *et al*., 2017). Yet, again, the pili samples used in these experiments are not pure. Indeed, AFM imaging revealed substantial contamination by ‘globular debris’, which the authors acknowledged could correspond to contaminating proteins, including c‐Cyts, and nonproteinaceous cell material (Ing *et al*., 2017). Furthermore, the crude preparation was spun‐coated onto the electrodes, a deposition approach that subjects the sample to sequential steps of dehydration.

The proposal that GS T4P can function as metallic wires is provocative and reminiscent of the first descriptions of DNA molecules as ‘pi‐way’ conductors or ‘wires’ (Turro and Barton, 1998). It is widely accepted now that long‐range charge transport in DNA occurs primarily via a multistep hopping mechanism in which guanine base pairs function as carriers of the positive charge (Giese, 2002). Conflicting interpretations about the nature of DNA conductivity have been linked to the sensitivity of transport measurements to the purity of the samples, intrinsic properties of the DNA molecule (such as conformation and length), and extrinsic parameters that modulate these properties (e.g., humidity) (Yamahata *et al*., 2008). Evidence is also emerging in support for a mechanism of GS T4P conductance dominated by multistep hopping (Feliciano *et al*., 2015). Single molecule conductivity measurements have been particularly useful to investigate how the GS T4P protein matrix permits long‐range electron transfer in the absence of inorganic and organic cofactors (Lampa‐Pastirk *et al*., 2016). Charge transport along individual T4P fibres probed without substantial dehydration is, for example, thermally activated at biological potentials (100–200 mV), a hallmark of incoherent redox conductivity (Lampa‐Pastirk *et al*., 2016). A similar thermal dependence has been demonstrated for living electroactive biofilms (Yates *et al*., 2015), which are permeated by the conductive T4P network to maintain optimal rates of charge transport and biofilm growth (Steidl *et al*., 2016). Further supporting the hopping model of T4P conductance, the carrier mobility of individual fibres (3.2 × 10^−2^ cm^2^ V^−1^ s^−1^) (Lampa‐Pastirk *et al*., 2016) is orders of magnitude lower than the mobilities (> 1 cm^2^ V^−1^ s^−1^) that would be needed to describe pilus charge transport according to band theory (Polizzi *et al*., 2012).

**Figure 4:**
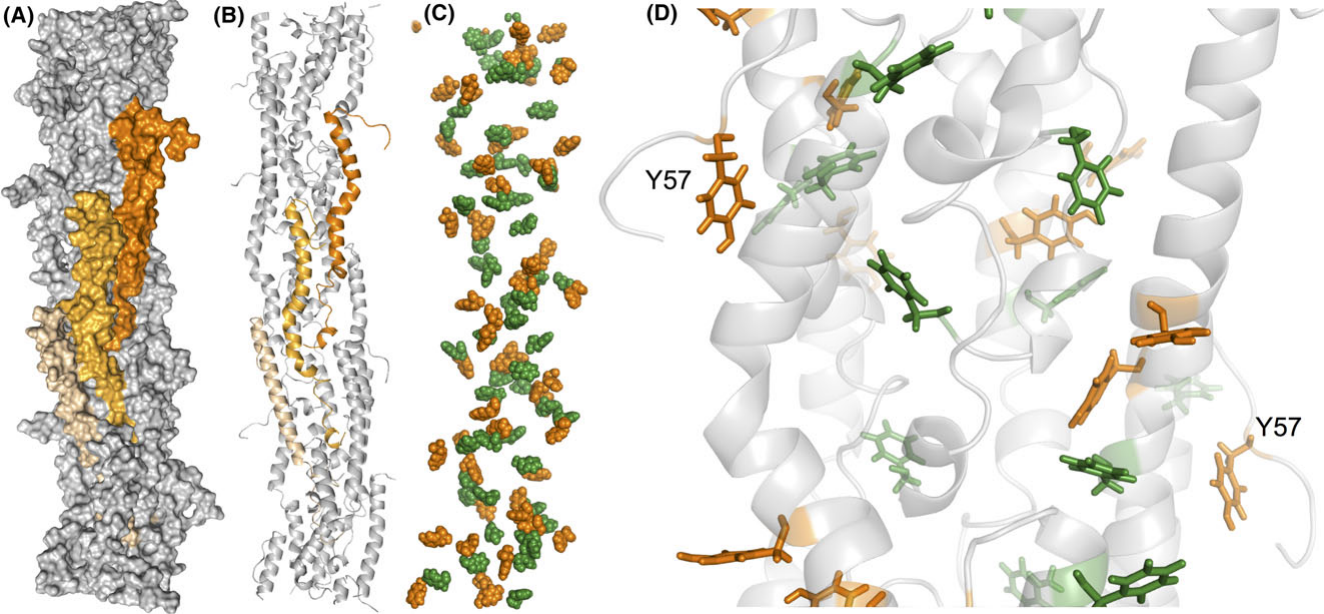
(A‐B) Surface map (A) and molecular structure (B) of a pilus fibre optimized via molecular dynamics showing in shades of orange three neighbouring pilins. (C) Clustering of aromatic residues (tyrosines, orange; phenylalanines, green) in the pilus fibre to form axial and transversal paths for electron transfer. (D) 20‐Å close up of a pilus region showing the parallel‐displaced and T‐shaped geometric configurations of the aromatic contacts (tyrosines, orange; phenylalanines, green). The tyrosine (Y57) exposed on each pilin's flexible carboxyl‐terminal segment is labelled.

Evidence for π–π stacking of aromatic side‐chains in the pilus fibres, as proposed in the metallic model, is also lacking. In the absence of experimental verification of the T4P structure, insights into interaromatic distances and the configuration of the aromatic side‐chains have been gained from computational models built using homologous T4P structures (Bonanni *et al*., 2013; Reardon and Mueller, 2013; Feliciano *et al*., 2015; Malvankar *et al*., 2015; Xiao *et al*., 2016). Although the distribution of aromatic residues in these models varies, none have the sandwich‐type dimer geometries that are needed to allow for π‐π orbital stacking and metallic conductivity. Only one of these models has been optimized in molecular dynamics (MD) to improve the accuracy of the structural predictions (Feliciano *et al*., 2015). Structural assumptions in this model were minimized using experimentally validated structures of the GS pilin (Reardon and Mueller, 2013) and the T4P template (the *Neisseria ghonorrhoeae* T4P) (Craig *et al*., 2006). The homology model was then subjected to MD simulations in solution to refine it to higher resolution, reduce uncertainties and mimic the *in‐vivo* solvation and dynamics of the *Geobacter* T4P. Furthermore, the model was tested experimentally to confirm its predictive value. Figure 4 shows a snapshot of the structure of the MD‐optimized GS fibre and the paths of aromatic side‐chains predicted to transport charges axially and transversally (Feliciano *et al*., 2015). Some aromatic rings in these paths cluster at distances between 3.5 and 5 Å but the aromatic contacts do not form at the same time, as in a wire. Furthermore, the geometric configuration of the contacts is of the parallel‐displaced or T‐shaped type, which is too disorganized to provide the type of charge delocalization expected for a metallic conductor (Feliciano *et al*., 2015). Energy‐minimized models, although nondynamic and rigid and, therefore, more prone to clustering artifacts, have reproduced similar geometric configurations as well (Xiao *et al*., 2016). The lack of perfectly aligned aromatic geometries in all the T4P models is not unexpected considering the substantial energy investment that would be required to maintain such rigid configurations. The formation of sandwich‐type aromatic dimers will also require a matrix environment with minimal molecular motions, as even small displacements decrease tunnelling rates through the stacked aromatic rings significantly (Bredas *et al*., 2002). It is difficult to envision how these fluctuations could be prevented in T4P, which are built with flexible, helical peptides (Fig. 4) to accommodate to the antagonistic cycles of fibre extension and retraction that they undergo *in vivo* (Fig. 2). Yet, these very same dynamic features could promote and even accelerate *in‐vivo* transport via multistep hopping.

The physiological relevance of the hopping mechanism also needs to consider how aromatic side‐chains can function as relay stations at the low biological potentials that operate *in vivo*, as demonstrated for a number of other proteins (Bennati *et al*., 2003; Stubbe, 2003; Stubbe *et al*., 2003; Yee *et al*., 2003). The MD model of the GS T4P predicts an electrostatic environment around the aromatic residues that could favour the transient protonation of acidic residues to reduce the oxidation potential of neighbouring tyrosines to the levels needed to function as relay amino acids *in vivo* (Feliciano *et al*., 2015). Such mechanism of proton‐coupled electron transfer tunes the redox activity of aromatic residues to enable fast rates of charge transport despite the low potentials that operate in biological systems (Stubbe *et al*., 2003; Reece and Nocera, 2009). Indeed, whereas the oxidization of tyrosine (TyrOH) to its radical cation (TyrO^•^H^+^) in water would require a strong oxidant (*E* ^0^ = 1.44 V vs. Normal Hydrogen Electrode [NHE]), deprotonation of the tyrosine to tyrosinate (TyrO^–^) reduces the oxidation potential in half (*E* ^0^ = 0.71 V vs. NHE) (Hammarstrom and Styring, 2011). In the pili, these electrochemical potentials are further modulated by the acidic and basic side‐chains from neighbouring amino acids, which function as proton acceptors and donors for the tyrosine respectively.

The sequential or simultaneous transfer of a proton and electron also makes this mechanism of charge hopping sensitive to the local pH. The proton‐coupled potential of tyrosine decreases, for example, in 59 mV increments for each pH unit changed between −2 and 10 (Hammarstrom and Styring, 2011). This is particularly relevant in electroactive biofilms, where the pH is lower in the region closer to the electrode than in the upper stratum (Franks *et al*., 2009; Babauta *et al*., 2012). As a result, a pH gradient is established that has been proposed to tune the rates of pilus charge transport and electronic interactions with the matrix‐associated c‐Cyts (Steidl *et al*., 2016). This may enable the mechanistic stratification of the biofilms, with T4P and c‐Cyts operating coordinately to transfer electrons in the more acidic layers closer to the electrode (Steidl *et al*., 2016). Yet, as the biofilms grow in thickness, the pH gradient dissipates and the T4P progressively become the primary mechanism for charge transport (Steidl *et al*., 2016). This is also the upper region of the biofilm where diffusion constraints limit the rate of electron transfer via c‐Cyts, making electron discharges via the T4P critical to maintain optimal growth of the cells via respiration (Steidl *et al*., 2016).

### Metal trapping and reduction at the pilus surface

**Figure 5:**
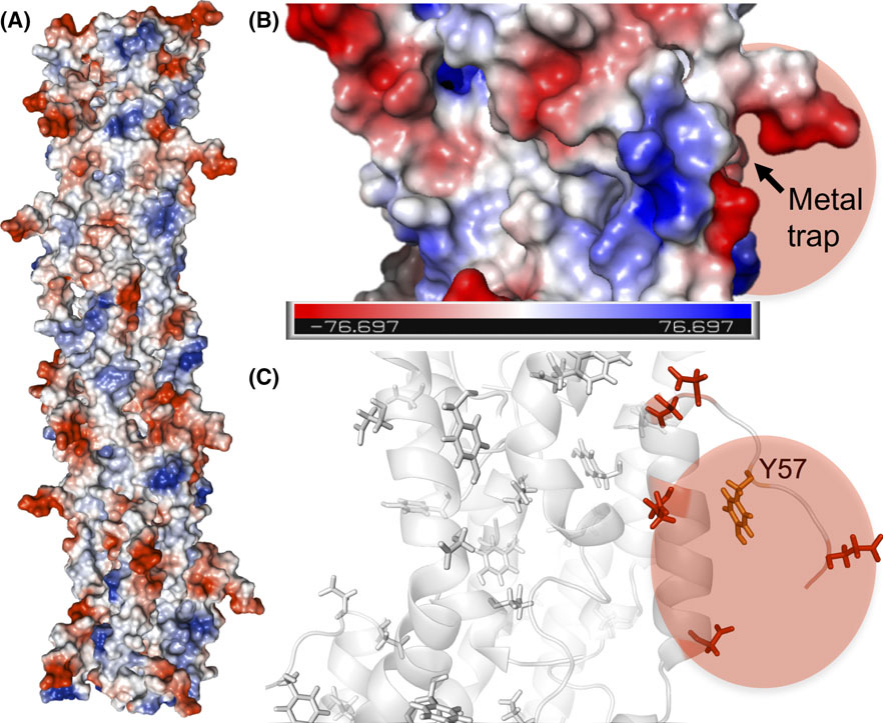
(A‐B) Electrostatic potential (blue, positive; red, negative) map of a pilus fibre (A) and a 20‐Å closeup of a region with a metal trap (B). Colour bar in (B), Volts. (C) Molecular structure of the region around the metal trap highlighting in red the acidic side‐chains and, in orange, the terminal tyrosine (Y57) of the transversal electron transfer path.

The ability of the conductive T4P to strongly bind the soluble uranyl cation and reduce it to a mineral form (Cologgi *et al*., 2011) suggests that ligands exist on the pilus surface that function as metal traps. Furthermore, these surface motifs must be close to exposed aromatic residues to permit the transfer of electrons and the reduction of the bound metal. The atomic environment modelled from T4P‐bound uranium L_III_‐edge Extended X‐ray Absorption Spectroscopy spectra is consistent with a metal trap containing two carboxyl ligands in opposite orientations (Cologgi *et al*., 2011). The MD model of the T4P fibre shows most of the negatively charged chains concentrated on the pilus surface and in regions surrounding the solvent‐exposed carboxy‐terminal segment of each pilin in the assembly (Feliciano *et al*., 2015). These surface motifs provide several acidic pair combinations suitable for metal trapping and include one of the carboxyl ligands of the pilin's random coil segment, which has the flexibility and exposure needed to promote metal binding (Fig. 5). The caging of uranium by these carboxyl ligands also positions the metal atom close to the most exposed tyrosine (Y57), which is predicted to catalyse the last step in electron transfer to extracellular electron acceptors (Feliciano *et al*., 2015). The spatial distance between the caged metal and the tyrosine is less than 2 nm, a distance optimal for tunnelling (Gray and Winkler, 2005). The proximity of the carboxyl ligands to the tyrosine also favours its deprotonation, which could reduce the oxidation potential of the aromatic side‐chain to promote fast rates of electron transfer to the pilus‐bound metal via a proton‐coupled hopping mechanism (Stubbe *et al*., 2003; Reece and Nocera, 2009).

In the MD model of the conductive T4P, most of the positively charged chains are buried in the pilus fibre core (Feliciano *et al*., 2015), a surface property that enhances the binding capacity of the metal traps (Fig. 5). The surface of the T4P contains but a few, small positively charged regions surrounded by regions of neutral charge, a configuration that minimizes electron trapping (Feliciano *et al*., 2015). This electrostatic surface distribution is expected to favour the axial flow of electrons through the aromatic contacts in the fibre's inner core until the acidic ligands in the trap bind the cationic metal. At this point, the surface negative charges are neutralized, permitting the transversal flow of electrons to the exposed tyrosine (Y57) and the reduction of the bound metal.

## Synthetic biology goes electronic

### Harnessing pilin self‐assembly in hybrid electronic devices

**Figure 6:**
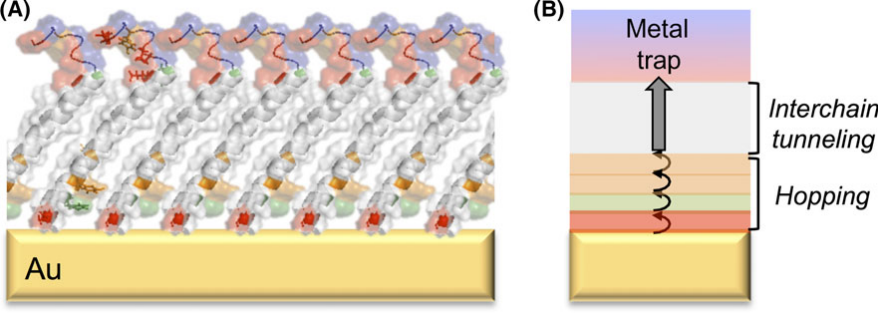
Gold (Au) electrodes functionalized with conductive pilin monolayers. (A) Predicted structure of a monolayer of truncated pilins attached to the electrode with a cysteine tag (red) and showing the aromatic amino acids (phenylalanine, green; tyrosines, orange) and acidic ligands (red) of the metal traps exposed to the solvent. (B) Cartoon illustrating the favoured flow of electrons from the aromatic doped region dominated by charge hopping, across the region of interchain tunnelling and to the metal trap.

The complex electronic properties that result from the tight and helical assembly of the *Geobacter* pilins and nanowire dimensions could allow for hopping regimes to operate *in vivo* along with other modes of conduction. Some regions of the GS T4P are aromatic free yet bring adjacent helices sufficiently close to each other to support interchain tunnelling (Fig. 4). This mechanism of hybrid conduction has been demonstrated in planar assemblies of GS pilin derivatives produced via recombinant techniques (Cosert *et al*., 2017) and is analogous to the spatial transition between tunnelling and hopping regimes that allows ‘tour wires’ (molecular wires of oligo(*p*‐phenylene ethynylene) and derivatives) to efficiently transport charges throughout long distances (Lu *et al*., 2009). The recombinant GS pilins carry a truncation in the first 19 amino acids, which are needed for *in‐vivo* assembly, but retain most of the α1 domain that is needed for self‐assembly via hydrophobic interactions (Fig. 6). Once engineered with an amino‐terminal cysteine tag, the peptides self‐assemble as an ordered and dense monolayer attached to gold electrodes (Cosert *et al*., 2017). The truncated peptides carry all of the aromatic residues needed for electronic coupling and charge transport in the T4P (Feliciano *et al*., 2015). Yet, in the planar peptide assembly, the aromatic rings cluster closer to the electrode, vertically stratifying the monolayers as an aromatic‐rich bottom stratum and an aromatic‐free zone of tightly packed helices in the upper region of the assembly (Fig. 6). On the other hand, the metal traps are concentrated on top of the monolayer, exposed to the solvent (Fig. 6).

The pilin monolayers are conductive and display the distance response (weakly dependent and exponential) of hopping and tunnelling respectively (Cosert *et al*., 2017) (Fig. 6). The crossover from the hopping to the tunnelling mechanism matches the spatial transition from aromatic‐rich to aromatic‐free regions in the monolayer. In addition, the concentration of aromatic side‐chains closer to the electrode effectively ‘dopes’ the pilins at the amino‐terminus and leads to voltage‐dependent rectification whereby the upward flow of electrons (towards the metal traps exposed to the solvent) is favoured at low (100 mV) voltages (Cosert *et al*., 2017). Rectification is likely enhanced by the positively charged side‐chains that surround the aromatic residues in this region, as they can locally compress the helix to modulate the interaromatic distances as a function of the applied voltage (Cosert *et al*., 2017). Genetic engineering could be used to tune the rectification, dopant effect and metal binding affinity of the monolayers to develop sensors and deployable devices for bioremediation and biomining. Because the stratification of aromatic‐free and rich regions in the monolayers mirrors the distribution of aromatics in the T4P, a similar hybrid mechanism may operate in the T4P to promote long‐range charge transport (Lampa‐Pastirk *et al*., 2016). This suggests that genetic engineering approaches such as those proposed for the pilin monolayers could also be applied to modulate the material's properties of protein nanowires for niche applications.

### Industrial microbiology wires up

The conductive T4P also promote interactions leading to cell–cell aggregation (Fig. 1) (Reguera *et al*., 2007) and formation of electroactive biofilms (Reguera *et al*., 2006, 2007). The ability of biofilms to couple growth to current generation depends on the ability of the T4P to conduct electrons (Vargas *et al*., 2013; Feliciano *et al*., 2015) and stabilize and support the biofilm matrix as the cells grow away from the electrode (Reguera *et al*., 2007). The stabilization of bacterial biofilm matrices has been proposed to involve electrostatic interactions between T4P and other matrix‐associated components (Chen and Stewart, 2002; Flemming and Wingender, 2010). Consistent with this, biofilms of an Asp2 mutant, which produces pili with alanine replacements in two acidic residues of the pilin to neutralize the net negative charge of the pilus surface charge, are delayed in current initiation by almost 12 h and reach half the current yields of the wild‐type (Feliciano *et al*., 2015). This phenotype is similar to the phenotype of mutant biofilms that express Tyr3 T4P (Feliciano *et al*., 2015), which carry alanine replacements in the pilin's three tyrosines that increase the electrical resistance of the assembled fibres fivefold (Steidl *et al*., 2016). This suggests that the discharge of respiratory electrons by biofilm cells depends on both the conductivity of the T4P and their ability to establish productive interactions with matrix‐associated c‐Cyts (Steidl *et al*., 2016).

As biofilms grow on the electrode, they reach a threshold thickness of about 10 μm that limits the diffusion of the c‐Cyts and their ability to transport charges. This leads to the progressive accumulation of reduced c‐Cyts in the biofilm matrix (Liu and Bond, 2012) and the generation of a redox gradient that provides the driving force for electrons to flow towards the underlying electrode (Snider *et al*., 2012). To alleviate the electron–acceptor limitation imposed by the c‐Cyts, cells in this upper stratum use the conductive T4P network as the primary path for electron discharges (Steidl *et al*., 2016). The dynamics of the T4P are also important in the biofilm matrix. Antagonistic cycles of protrusion and retraction have been proposed to allow the cells to coordinate interactions with matrix‐associated c‐Cyts and to modulate cell–cell distances, so nutrient diffusion and charge transport through the biofilms is not limited (Speers *et al*., 2016). In support of this, mutants unable to retract the T4P are hyperpiliated, aggregate tightly and form denser biofilms on the electrodes, yet the rates of charge discharge per cell are reduced compared to the wild type (Speers *et al*., 2016).

By allowing the cells to continue to grow at a distance from the anode electrode, the T4P promote the harvesting of current from the oxidation of electron donors and proportionally to the biofilm thickness (Reguera *et al*., 2006). GS biofilms grown on anode electrodes (bioanodes) can harvest current from the oxidization of common fermentation byproducts such as acetate, formate and lactate, but only acetate is efficiently assimilated for carbon and/or oxidized by the biofilms (Speers and Reguera, 2012a,b). Genetic engineering has been successfully applied to develop bioanodes with greater tolerance to alcohol products and more efficient metabolism of the electron donors (Speers *et al*., 2014; Awate *et al*., 2017). These improvements permitted the operation of the bioanodes as ‘scrubbers’ of unwanted fermentation products produced during ethanologenic fermentations (Awate *et al*., 2017). Inactivating hydrogen uptake in the bioanode cells also makes possible the operation of commercial reactors as a single‐chambered microbial electrolysis cell, an electrochemical system that uses the electricity harvested at the bioanode to produce hydrogen fuel at the cathode (Awate *et al*., 2017). Powering fermentations with bioanodes (anodic electro‐fermentations) prevents the accumulation of feedback inhibitors in the broth, stimulates the rates of fermentation and increases product titres and productivity (Speers and Reguera, 2012b; Awate *et al*., 2017). Anodic electro‐fermentations can also prevent pH imbalances, reduce batch‐to‐batch variability and increase product quality (Awate *et al*., 2017). As a result, the value‐added product is enriched in the fermentation broth, reducing the steps and costs associated with its downstream purification. The genetic amenability of the bioanodes thus offers opportunities to custom‐tailor their activities for syntrophic interactions with specific fermentation partners and develop electro‐fermentations with the economics needed for industrial implementation.

## Conclusions and outlook

The ability of a peptide assembly such as the GS T4P to transport electrons at μm distances is especially significant for applications in nanotechnology. The demonstration that recombinant techniques can be applied to synthesize conductive pilin derivatives (Cosert *et al*., 2017) shows promise to develop sustainable manufacturing protocols for the mass‐production of pilin‐inspired peptides. These derivatives can be functionalized via genetic engineering to modulate the specificity of their interactions with substrates, which could enable simple protocols for the manufacturing of peptide arrays with multiplex functionality for sensing applications.

Information about the amino acid residues and structural features of the pilin that contribute to its self‐assembly also merits investigation as it could be used to promote the self‐assembly of the peptide building blocks in a cell‐free environment and manufacture protein nanowires *in vitro*. Recombinant peptides could be designed to modulate the morphology of the supramolecular assembly and tune the optical and conductive properties of the material (Tao *et al*., 2017). Knowledge about the amino acids that mediate the conductivity is also critical to manipulate the conductive properties of the peptides and assemblies. Moreover, the functionalization of the peptides via genetic engineering could be harnessed to add chemical tags for the selective and specific integration of the nanowires into electronic devices. The ability to design and mass‐produce generations of protein nanowires using recombinant peptides as building blocks and their assembly in a cell‐free environment contrasts with the harsh protocols and specialized equipment that is traditionally needed to fabricate inorganic nanowires (Long *et al*., 2012) and the challenges that still remain for their functionalization (Cui *et al*., 2001; Fennell *et al*., 2016). Conductive peptides and protein nanowires also circumvent major concerns about the toxicity of many conductive nanomaterials, which could facilitate their commercial implementation (Love *et al*., 2012). Thus, novel applications could be envisioned that capitalize on the biocompatibility of these novel materials in nanoelectronics and nanomedicine.

Of special interest for bioremediation applications is the natural ability of *Geobacter* pili to bind and reductively precipitate cationic metal contaminants such as the uranyl cation (Cologgi *et al*., 2011). Sensors could be developed that harness the planar self‐assembly of pilin derivatives, their conductivity and concentration of metal traps on the surface to increase the sensitivity of detection (Cosert *et al*., 2017). Because metal binding is mediated by acidic amino acid ligands, genetic engineering could be used to fine‐tune the binding kinetics and specificity and reach the selectivity needed for environmental sensing applications. The ability to mass‐produce recombinant pilin peptides that retain their metal trap (Cosert *et al*., 2017) also opens opportunities to functionalize inexpensive materials suitable for deployment and *in‐situ* immobilization of metal contaminants. Such platforms could enable the bioremediation of environments impacted by a wide range of cationic metals such as uranium, cadmium and cobalt. Because metal trapping is mediated by electrostatic interactions, the same or similar platforms could also be employed for the reclamation of precious and rare metals such as gold, silver and lanthanides.

The role of *Geobacter* T4P as electron carriers in current‐harvesting biofilms is also significant for a number of energy‐related applications (Borole *et al*., 2011). The conductive pili permeate the biofilms and electronically connect the cells to other electron carriers of the biofilm matrix (e.g. c‐Cyts) and the underlying electrode (Steidl *et al*., 2016). Understanding the interactions between pili and matrix‐associated c‐Cyts could provide strategies to optimize current harvesting by biofilms, particularly in the lower biofilm stratum (~ 10‐μm thick) where these electron carriers cooperate to maintain optimum rates of electron transfer. As the biofilms grow in thickness, the ability of the c‐Cyts to efficiently transport charges decreases and the pili become the primary mechanism for electron transfer by the biofilm cells (Steidl *et al*., 2016). Genetic manipulation of the number of T4P produced per cell or their rates of charge transport could effectively alleviate the electron acceptor limitation imposed by the accumulation of reduced c‐Cyts in the top layers of thick biofilms and improve the performance of bioanodes. In addition to stacking more cells on electrodes, the GS pili and, to a lesser extent, matrix‐associated c‐Cyts, enhance the capacity of cells in biofilms to bind and reduce soluble, toxic metals such as the uranyl cation (Cologgi *et al*., 2014), a catalytic activity that could be harnessed to develop permeable biobarriers for the *in‐situ* bioremediation of metal contaminants.

Harnessing the power of electroactive biofilms also needs to consider strategies to improve the metabolic versatility of the biofilm cells. Bioanodes show promise as scrubbers of fermentation inhibitors (Speers and Reguera, 2012a,b; Speers *et al*., 2014; Awate *et al*., 2017). Thus, they could be scaled up for their integration into industrial fermenters (electro‐fermentations). Anodic electro‐fermentations circumvent many of the limitations that prevent the implementation of otherwise attractive fermentation‐based technologies at the industrial level. The success of these technologies will ultimately depend on our ability to manufacture bioanodes with the robustness and scales needed for operation in industrial fermentations. Lower scales may also be considered to design decentralized electro‐fermentation platforms that harness the syntrophic interactions of fermentative bacteria with bioanodes to treat a wide range of wastewaters and promote water reuse. Environmental studies of mixed species biofilms could advance these applications greatly. Fe(III) oxide minerals are abundant in soils and sediments and provide both an electron acceptor and a surface for the growth of electroactive biofilms. These biofilms are integral components of the consortia that participate in the decomposition of organic matter and the cycling of metals, including toxic metals. The interactions that drive the functioning of these environmental consortia could inspire the design of synthetic electroactive communities and novel bioenergy and bioremediation platforms to harvest energy from waste organic matter and immobilize contaminants.

## Conflict of interest

The author is founder and Chief Scientific Officer of BioElectrica Inc., a startup that uses microorganisms to advance waste to energy technologies.

## References

[mbt213280-bib-0001] Alfaro‐Aco, R. , and Petry, S. (2015) Building the microtubule cytoskeleton piece by piece. J Biol Chem 290: 17154–17162. 25957410 10.1074/jbc.R115.638452 PMC4498055

[mbt213280-bib-0002] Audette, G. F. , van Schaik, E. J. , Hazes, B. , and Irvin, R. T. (2004a) DNA‐binding protein nanotubes: learning from Nature's nanotech examples. Nano Lett 4: 1897–1902.

[mbt213280-bib-0003] Audette, G. F. , Irvin, R. T. , and Hazes, B. (2004b) Crystallographic analysis of the *Pseudomonas aeruginosa* strain K122‐4 monomeric pilin reveals a conserved receptor‐binding architecture. Biochemistry 43: 11427–11435. 15350129 10.1021/bi048957s

[mbt213280-bib-0004] Awate, B. , Steidl, R. J. , Hamlischer, T. , and Reguera, G. (2017) Stimulation of electro‐fermentation in single‐chamber microbial electrolysis cells driven by genetically engineered anode biofilms. J Power Sources 356: 510–518.

[mbt213280-bib-0005] Babauta, J. T. , Nguyen, H. D. , Harrington, T. D. , Renslow, R. , and Beyenal, H. (2012) pH, redox potential and local biofilm potential microenvironments within *Geobacter sulfurreducens* biofilms and their roles in electron transfer. Biotechnol Bioeng 109: 2651–2662. 22549331 10.1002/bit.24538 PMC3551578

[mbt213280-bib-0006] Bennati, M. , Weber, A. , Antonic, J. , Perlstein, D. L. , Robblee, J. , and Stubbe, J. (2003) Pulsed ELDOR spectroscopy measures the distance between the two tyrosyl dadicals in the R2 subunit of the *E. coli* ribonucleotide reductase. J Am Chem Soc 125: 14988–14989. 14653724 10.1021/ja0362095

[mbt213280-bib-0007] Bertone, P. , and Snyder, M. (2005) Advances in functional protein microarray technology. FEBS J 272: 5400–5411. 16262682 10.1111/j.1742-4658.2005.04970.x

[mbt213280-bib-0008] Bonanni, P. S. , Massazza, D. , and Busalmen, J. P. (2013) Stepping stones in the electron transport from cells to electrodes in *Geobacter sulfurreducens* biofilms. Phys Chem Chem Phys 15: 10300–10306. 23698325 10.1039/c3cp50411e

[mbt213280-bib-0009] Borole, A. P. , Reguera, G. , Ringeisen, B. , Wang, Z.‐W. , Feng, Y. , and Kim, B. H. (2011) Electroactive biofilms: Current status and future research needs. Energy Environ Sci 4: 4813–4834.

[mbt213280-bib-0010] Bredas, J. L. , Calbert, J. P. , da Silva Filho, D. A. , and Cornil, J. (2002) Organic semiconductors: a theoretical characterization of the basic parameters governing charge transport. Proc Natl Acad Sci USA 99: 5804–5809. 11972059 10.1073/pnas.092143399 PMC122857

[mbt213280-bib-0011] Butler, J. E. , Young, N. D. , and Lovley, D. R. (2010) Evolution of electron transfer out of the cell: comparative genomics of six *Geobacter* genomes. BMC Genom 11: 40. 10.1186/1471-2164-11-40 PMC2825233 20078895

[mbt213280-bib-0012] Chen, X. , and Stewart, P. S. (2002) Role of electrostatic interactions in cohesion of bacterial biofilms. Appl Microbiol Biotechnol 59: 718–720. 12226730 10.1007/s00253-002-1044-2

[mbt213280-bib-0013] Chen, C. S. , Mrksich, M. , Huang, S. , Whitesides, G. M. , and Ingber, D. E. (1997) Geometric control of cell life and death. Science 276: 1425–1428. 9162012 10.1126/science.276.5317.1425

[mbt213280-bib-0014] Clausen, M. , Jakovljevic, V. , Sogaard‐Andersen, L. , and Maier, B. (2009) High‐force generation is a conserved property of type IV pilus systems. J Bacteriol 191: 4633–4638. 19429611 10.1128/JB.00396-09 PMC2704717

[mbt213280-bib-0015] Cologgi, D. L. , Lampa‐Pastirk, S. , Speers, A. M. , Kelly, S. D. , and Reguera, G. (2011) Extracellular reduction of uranium via *Geobacter* conductive pili as a protective cellular mechanism. Proc Natl Acad Sci USA 108: 15248–15252. 21896750 10.1073/pnas.1108616108 PMC3174638

[mbt213280-bib-0016] Cologgi, D. L. , Speers, A. M. , Bullard, B. A. , Kelly, S. D. , and Reguera, G. (2014) Enhanced uranium immobilization and reduction by *Geobacter sulfurreducens* biofilms. Appl Environ Microbiol 80: 6638–6646. 25128347 10.1128/AEM.02289-14 PMC4249037

[mbt213280-bib-0017] Cosert, K. M. , Steidl, R. J. , Castro‐Forero, A. , Worden, R. M. , and Reguera, G. (2017) Electronic characterization of *Geobacter sulfurreducens* pilins in self‐assembled monolayers unmasks tunnelling and hopping conduction pathways. Phys Chem Chem Phys 19: 11163–11172. 28402361 10.1039/c7cp00885f

[mbt213280-bib-0018] Craig, L. , and Li, J. (2008) Type IV pili: paradoxes in form and function. Curr Opin Struct Biol 18: 267–277. 18249533 10.1016/j.sbi.2007.12.009 PMC2442734

[mbt213280-bib-0019] Craig, L. , Taylor, R. K. , Pique, M. E. , Adair, B. D. , Arvai, A. S. , Singh, M. , *et al* (2003) Type IV pilin structure and assembly: X‐ray and EM analyses of *Vibrio cholerae* toxin‐coregulated pilus and *Pseudomonas aeruginosa* PAK pilin. Mol Cell 11: 1139–1150. 12769840 10.1016/s1097-2765(03)00170-9

[mbt213280-bib-0020] Craig, L. , Volkmann, N. , Arvai, A. S. , Pique, M. E. , Yeager, M. , Egelman, E. H. , and Tainer, J. A. (2006) Type IV pilus structure by cryo‐electron microscopy and crystallography: implications for pilus assembly and functions. Mol Cell 23: 651–662. 16949362 10.1016/j.molcel.2006.07.004

[mbt213280-bib-0021] Cui, Y. , Wei, Q. , Park, H. , and Lieber, C. M. (2001) Nanowire nanosensors for highly sensitive and selective detection of biological and chemical species. Science 293: 1289–1292. 11509722 10.1126/science.1062711

[mbt213280-bib-0022] El‐Naggar, M. Y. , Wanger, G. , Leung, K. M. , Yuzvinsky, T. D. , Southam, G. , Yang, J. , *et al* (2010) Electrical transport along bacterial nanowires from *Shewanella oneidensis* MR‐1. Proc Natl Acad Sci USA 107: 18127–18131. 20937892 10.1073/pnas.1004880107 PMC2964190

[mbt213280-bib-0023] Esteve‐Nunez, A. , Sosnik, J. , Visconti, P. , and Lovley, D. R. (2008) Fluorescent properties of *c*‐type cytochromes reveal their potential role as an extracytoplasmic electron sink in *Geobacter sulfurreducens* . Environ Microbiol 10: 497–505. 18093163 10.1111/j.1462-2920.2007.01470.x

[mbt213280-bib-0024] Feliciano, G. T. , da Silva, A. J. R. , Reguera, G. , and Artacho, E. (2012) The molecular and electronic structure of the peptide subunit of *Geobacter sulfurreducens* conductive pili from first principles. J Phys Chem A 116: 8023–8030. 22779741 10.1021/jp302232p

[mbt213280-bib-0025] Feliciano, G. T. , Steidl, R. J. , and Reguera, G. (2015) Structural and functional insights into the conductive pili of *Geobacter sulfurreducens* revealed in molecular dynamics simulations. Phys Chem Chem Phys 17: 22217–22226. 26243427 10.1039/c5cp03432a

[mbt213280-bib-0026] Fennell, J. F. Jr , Liu, S. F. , Azzarelli, J. M. , Weis, J. G. , Rochat, S. , Mirica, K. A. , *et al* (2016) Nanowire chemical/biological sensors: status and a roadmap for the future. Angew Chem Int Ed Engl 55: 1266–1281. 26661299 10.1002/anie.201505308

[mbt213280-bib-0027] Flemming, H. C. , and Wingender, J. (2010) The biofilm matrix. Nat Rev Microbiol 8: 623–633. 20676145 10.1038/nrmicro2415

[mbt213280-bib-0028] Franks, A. E. , Nevin, K. P. , Jia, H. , Izallalen, M. , Woodard, T. L. , and Lovley, D. R. (2009) Novel strategy for three‐dimensional real‐time imaging of microbial fuel cell communities: monitoring the inhibitory effects of proton accumulation within the anode biofilm. Energy Environ Sci 2: 113–119.

[mbt213280-bib-0029] Friedrich, C. , Bulyha, I. , and Sogaard‐Andersen, L. (2014) Outside‐in assembly pathway of the type IV pilus system in *Myxococcus xanthus* . J Bacteriol 196: 378–390. 24187092 10.1128/JB.01094-13 PMC3911261

[mbt213280-bib-0030] Giese, B. (2002) Long‐distance electron transfer through DNA. Annu Rev Biochem 71: 51–70. 12045090 10.1146/annurev.biochem.71.083101.134037

[mbt213280-bib-0031] Giltner, C. L. , Nguyen, Y. , and Burrows, L. L. (2012) Type IV pilin proteins: versatile molecular modules. Microbiol Mol Biol Rev 76: 740–772. 23204365 10.1128/MMBR.00035-12 PMC3510520

[mbt213280-bib-0032] Gorby, Y. A. , Yanina, S. , McLean, J. S. , Rosso, K. M. , Moyles, D. , Dohnalkova, A. , *et al* (2006) Electrically conductive bacterial nanowires produced by *Shewanella oneidensis* strain MR‐1 and other microorganisms. Proc Natl Acad Sci USA 103: 11358–11363. 16849424 10.1073/pnas.0604517103 PMC1544091

[mbt213280-bib-0033] Gray, H. B. , and Winkler, J. R. (2005) Long‐range electron transfer. Proc Natl Acad Sci USA 102: 3534–3539. 15738403 10.1073/pnas.0408029102 PMC553296

[mbt213280-bib-0034] Gupta, S. , Manubhai, K. P. , Kulkarni, V. , and Srivastava, S. (2016) An overview of innovations and industrial solutions in Protein Microarray Technology. Proteomics 16: 1297–1308. 27089056 10.1002/pmic.201500429

[mbt213280-bib-0035] Hammarstrom, L. , and Styring, S. (2011) Proton‐coupled electron transfer of tyrosines in Photosystem II and model systems for artificial photosynthesis: the role of a redox‐active link between catalyst and photosensitizer. Energy Environ Sci 4: 2379–2388.

[mbt213280-bib-0036] Hazes, B. , Sastry, P. A. , Hayakawa, K. , Read, R. J. , and Irvin, R. T. (2000) Crystal structure of *Pseudomonas aeruginosa* PAK pilin suggests a main‐chain‐dominated mode of receptor binding. J Mol Biol 299: 1005–1017. 10843854 10.1006/jmbi.2000.3801

[mbt213280-bib-0037] Holmes, D. E. , Dang, Y. , Walker, D. J. , and Lovley, D. R. (2016) The electrically conductive pili of *Geobacter* species are a recently evolved feature for extracellular electron transfer. Microb Genom 2: e000072. 28348867 10.1099/mgen.0.000072 PMC5320591

[mbt213280-bib-0038] Ing, N. L. , Nusca, T. D. , and Hochbaum, A. I. (2017) *Geobacter sulfurreducens* pili support ohmic electronic conduction in aqueous solution. Phys Chem Chem Phys 19: 21791–21799. 28783184 10.1039/c7cp03651e

[mbt213280-bib-0039] Jarrell, K. F. , and McBride, M. J. (2008) The surprisingly diverse ways that prokaryotes move. Nature Rev Microbiol 6: 466–476. 18461074 10.1038/nrmicro1900

[mbt213280-bib-0040] Juarez, K. , Kim, B. C. , Nevin, K. , Olvera, L. , Reguera, G. , Lovley, D. R. , and Methe, B. A. (2009) PilR, a transcriptional regulator for pilin and other genes required for Fe(III) reduction in *Geobacter sulfurreducens* . J Mol Microbiol Biotechnol 16: 146–158. 18253022 10.1159/000115849

[mbt213280-bib-0041] Karuppiah, V. , Collins, R. F. , Thistlethwaite, A. , Gao, Y. , and Derrick, J. P. (2013) Structure and assembly of an inner membrane platform for initiation of type IV pilus biogenesis. Proc Natl Acad Sci USA 110: E4638–E4647. 24218553 10.1073/pnas.1312313110 PMC3845092

[mbt213280-bib-0042] Kim, B. C. , and Lovley, D. R. (2008) Investigation of direct vs. indirect involvement of the *c*‐type cytochrome MacA in Fe(III) reduction by *Geobacter sulfurreducens* . FEMS Microbiol Lett 286: 39–44. 18616590 10.1111/j.1574-6968.2008.01252.x

[mbt213280-bib-0043] Kim, B. C. , Leang, C. , Ding, Y. H. , Glaven, R. H. , Coppi, M. V. , and Lovley, D. R. (2005) OmcF, a putative *c*‐type monoheme outer membrane cytochrome required for the expression of other outer membrane cytochromes in *Geobacter sulfurreducens* . J Bacteriol 187: 4505–4513. 15968061 10.1128/JB.187.13.4505-4513.2005 PMC1151787

[mbt213280-bib-0044] Kim, B. C. , Qian, X. , Leang, C. , Coppi, M. V. , and Lovley, D. R. (2006) Two putative *c*‐type multiheme cytochromes required for the expression of OmcB, an outer membrane protein essential for optimal Fe(III) reduction in *Geobacter sulfurreducens* . J Bacteriol 188: 3138–3142. 16585776 10.1128/JB.188.8.3138-3142.2006 PMC1447008

[mbt213280-bib-0045] Kotloski, N. J. , and Gralnick, J. A. (2013) Flavin electron shuttles dominate extracellular electron transfer by *Shewanella oneidensis* . MBio 4: e00553‐12. 23322638 10.1128/mBio.00553-12 PMC3551548

[mbt213280-bib-0046] Lampa‐Pastirk, S. , Veazey, J. P. , Walsh, K. A. , Steidl, R. J. , Tessmer, S. H. , and Reguera, G. (2016) Thermally activated charge transport in microbial protein nanowires. Sci Rep 6: 23517. 27009596 10.1038/srep23517 PMC4806346

[mbt213280-bib-0047] Lentini, C. J. , Wankel, S. D. , and Hansel, C. M. (2012) Enriched iron(III)‐reducing bacterial communities are shaped by carbon substrate and iron oxide mineralogy. Frontiers Microbiol 3: 404. 10.3389/fmicb.2012.00404 PMC3541049 23316187

[mbt213280-bib-0048] Liu, Y. , and Bond, D. R. (2012) Long‐distance electron transfer by *G. sulfurreducens* biofilms results in accumulation of reduced *c*‐type cytochromes. Chemsuschem 5: 1047–1053. 22577055 10.1002/cssc.201100734 PMC3500873

[mbt213280-bib-0049] Long, Y.‐Z. , Yu, M. , Sun, B. , Gu, C.‐Z. , and Fan, Z. (2012) Recent advances in large‐scale assembly of semiconducting inorganic nanowires and nanofibers for electronics, sensors and photovoltaics. Chem Soc Rev 41: 4560–4580. 22573265 10.1039/c2cs15335a

[mbt213280-bib-0050] Love, S. A. , Maurer‐Jones, M. A. , Thompson, J. W. , Lin, Y.‐S. , and Haynes, C. L. (2012) Assessing nanoparticle toxicity. Annu Rev Anal Chem 5: 181–205. 10.1146/annurev-anchem-062011-143134 22524221

[mbt213280-bib-0051] Lu, Q. , Liu, K. , Zhang, H. , Du, Z. , Wang, X. , and Wang, F. (2009) From tunneling to hopping: A comprehensive investigation of charge tansport mechanism in molecular junctions based on oligo(p‐phenylene ethynylene)s. ACS Nano 3: 3861–3868. 19916506 10.1021/nn9012687

[mbt213280-bib-0052] Ma, D. , Baruch, D. , Shu, Y. , Yuan, K. , Sun, Z. , Ma, K. , *et al* (2012) Using protein microarray technology to screen anti‐ERCC1 monoclonal antibodies for specificity and applications in pathology. BMC Biotechnol 12: 88. 23171216 10.1186/1472-6750-12-88 PMC3526464

[mbt213280-bib-0053] Maier, B. , and Wong, G. C. (2015) How bacteria use type IV pili machinery on surfaces. Trends Microbiol 23: 775–788. 26497940 10.1016/j.tim.2015.09.002

[mbt213280-bib-0054] Malvankar, N. S. , Vargas, M. , Nevin, K. P. , Franks, A. E. , Leang, C. , Kim, B. C. , *et al* (2011) Tunable metallic‐like conductivity in microbial nanowire networks. Nat Nanotechnol 6: 573–579. 21822253 10.1038/nnano.2011.119

[mbt213280-bib-0055] Malvankar, N. S. , Vargas, M. , Nevin, K. , Tremblay, P. L. , Evans‐Lutterodt, K. , Nykypanchuk, D. , *et al* (2015) Structural basis for metallic‐like conductivity in microbial nanowires. MBio 6: e00084. 25736881 10.1128/mBio.00084-15 PMC4453548

[mbt213280-bib-0056] Mattick, J. S. (2002) Type IV pili and twitching motility. Annu Rev Microbiol 56: 289–314. 12142488 10.1146/annurev.micro.56.012302.160938

[mbt213280-bib-0057] Morgado, L. , Bruix, M. , Pessanha, M. , Londer, Y. Y. , and Salgueiro, C. A. (2010) Thermodynamic characterization of a triheme cytochrome family from *Geobacter sulfurreducens* reveals mechanistic and functional diversity. Biophys J 99: 293–301. 20655858 10.1016/j.bpj.2010.04.017 PMC2895378

[mbt213280-bib-0058] Muruve, N. G. , Cheng, Y. F. , Feng, Y. , Liu, T. , Muruve, D. A. , Hassett, D. J. , and Irvin, R. T. (2016) Peptide‐based biocoatings for corrosion protection of stainless steel biomaterial in a chloride solution. Mater Sci Eng C Mater Biol Appl 68: 695–700. 27524070 10.1016/j.msec.2016.06.053

[mbt213280-bib-0059] Pelicic, V. (2008) Type IV pili: *e pluribus unum*? Mol Microbiol 68: 827–837. 18399938 10.1111/j.1365-2958.2008.06197.x

[mbt213280-bib-0060] Pessanha, M. , Morgado, L. , Louro, R. O. , Londer, Y. Y. , Pokkuluri, P. R. , Schiffer, M. , and Salgueiro, C. A. (2006) Thermodynamic characterization of triheme cytochrome PpcA from Geobacter sulfurreducens: Evidence for a role played in e‐/H+ energy transduction. Biochemistry 45: 13910–13917. 17105209 10.1021/bi061394v

[mbt213280-bib-0061] Pirbadian, S. , Barchinger, S. E. , Leung, K. M. , Byun, H. S. , Jangir, Y. , Bouhenni, R. A. , *et al* (2014) *Shewanella oneidensis* MR‐1 nanowires are outer membrane and periplasmic extensions of the extracellular electron transport components. Proc Natl Acad Sci USA 111: 12883–12888. 25143589 10.1073/pnas.1410551111 PMC4156777

[mbt213280-bib-0062] Polizzi, N. F. , Skourtis, S. S. , and Beratan, D. N. (2012) Physical constraints on charge transport through bacterial nanowires. Faraday Discuss 155: 43–62. 22470966 10.1039/c1fd00098e PMC3392031

[mbt213280-bib-0063] Reardon, P. N. , and Mueller, K. T. (2013) Structure of the type IVa major pilin from the electrically conductive bacterial nanowires of *Geobacter sulfurreducens* . J Biol Chem 288: 29260–29266. 23965997 10.1074/jbc.M113.498527 PMC3795228

[mbt213280-bib-0064] Reece, S. Y. , and Nocera, D. G. (2009) Proton‐coupled electron transfer in biology: results from synergistic studies in natural and model systems. Annu Rev Biochem 78: 673–699. 19344235 10.1146/annurev.biochem.78.080207.092132 PMC4625787

[mbt213280-bib-0065] Reguera, G. (2012) Electron transfer at the cell‐uranium interface in *Geobacter* spp. Biochem Soc Trans 40: 1227–1232. 23176459 10.1042/BST20120162

[mbt213280-bib-0066] Reguera, G. , McCarthy, K. D. , Mehta, T. , Nicoll, J. S. , Tuominen, M. T. , and Lovley, D. R. (2005) Extracellular electron transfer via microbial nanowires. Nature 435: 1098–1101. 15973408 10.1038/nature03661

[mbt213280-bib-0067] Reguera, G. , Nevin, K. P. , Nicoll, J. S. , Covalla, S. F. , Woodard, T. L. , and Lovley, D. R. (2006) Biofilm and nanowire production lead to increased current in microbial fuel cells. Appl Environ Microbiol 72: 7345–7348. 16936064 10.1128/AEM.01444-06 PMC1636155

[mbt213280-bib-0068] Reguera, G. , Pollina, R. B. , Nicoll, J. S. , and Lovley, D. R. (2007) Possible nonconductive role of *Geobacter sulfurreducens* pilus nanowires in biofilm formation. J Bacteriol 189: 2125–2127. 17158673 10.1128/JB.01284-06 PMC1855775

[mbt213280-bib-0069] Richter, L. V. , Sandler, S. J. , and Weis, R. M. (2012) Two isoforms of *Geobacter sulfurreducens* PilA have distinct roles in pilus biogenesis, cytochrome localization, extracellular electron transfer, and biofilm formation. J Bacteriol 194: 2551–2563. 22408162 10.1128/JB.06366-11 PMC3347174

[mbt213280-bib-0070] Richter, L. V. , Franks, A. E. , Weis, R. M. , and Sandler, S. J. (2017) Significance of a post‐translational modification of the PilA protein of *Geobacter sulfurreducens* for surface attachment, biofilm formation and growth on insoluble extracellular electron acceptors. J Bacteriol 199: e00716‐16. 28138101 10.1128/JB.00716-16 PMC5370424

[mbt213280-bib-0071] Ruhle, T. , and Leister, D. (2015) Assembly of F_1_F_0_‐ATP synthases. Biochim Biophys Acta 1847: 849–860. 25667968 10.1016/j.bbabio.2015.02.005

[mbt213280-bib-0072] Sanford, R. A. , Wu, Q. , Sung, Y. , Thomas, S. H. , Amos, B. K. , Prince, E. K. , and Loffler, F. E. (2007) Hexavalent uranium supports growth of *Anaeromyxobacter dehalogenans* and *Geobacter* spp. with lower than predicted biomass yields. Environ Microbiol 9: 2885–2893. 17922770 10.1111/j.1462-2920.2007.01405.x

[mbt213280-bib-0073] Shi, L. , Dong, H. , Reguera, G. , Beyenal, H. , Lu, A. , Liu, J. , *et al* (2016) Extracellular electron transfer mechanisms between microorganisms and minerals. Nat Rev Microbiol 14: 651–662. 27573579 10.1038/nrmicro.2016.93

[mbt213280-bib-0074] Shin, Y.‐G. K. , Newton, M. D. , and Isied, S. S. (2003) Distance dependence of electron transfer across peptides with different secondary structures: the role of peptide energetics and electronic coupling. J Am Chem Soc 125: 3722–3732. 12656602 10.1021/ja020358q

[mbt213280-bib-0075] Shu, C. , Xiao, K. , Yan, Q. , and Sun, X. (2016) Comparative analysis of Type IV pilin in *Desulfuromonadales* . Front Microbiol 7: 2080. 28066394 10.3389/fmicb.2016.02080 PMC5174107

[mbt213280-bib-0076] Sleytr, U. B. , Schuster, B. , Egelseer, E. M. , and Pum, D. (2014) S‐layers: principles and applications. FEMS Microbiol Rev 38: 823–864. 24483139 10.1111/1574-6976.12063 PMC4232325

[mbt213280-bib-0077] Snider, R. M. , Strycharz‐Glaven, S. M. , Tsoi, S. D. , Erickson, J. S. , and Tender, L. M. (2012) Long‐range electron transport in *Geobacter sulfurreducens* biofilms is redox gradient‐driven. Proc Natl Acad Sci USA 109: 15467–15472. 22955881 10.1073/pnas.1209829109 PMC3458377

[mbt213280-bib-0078] Speers, A. M. , and Reguera, G. (2012a) Electron donors supporting growth and electroactivity of *Geobacter sulfurreducens* anode biofilms. Appl Environ Microbiol 78: 437–444. 22101036 10.1128/AEM.06782-11 PMC3255729

[mbt213280-bib-0079] Speers, A. M. , and Reguera, G. (2012b) Consolidated bioprocessing of AFEX‐pretreated corn stover to ethanol and hydrogen in a microbial electrolysis cell. Environ Sci Technol 46: 7875–7881. 22697183 10.1021/es3008497

[mbt213280-bib-0080] Speers, A. M. , Young, J. M. , and Reguera, G. (2014) Fermentation of glycerol into ethanol in a microbial electrolysis cell driven by a customized consortium. Environ Sci Technol 48: 6350–6358. 24802954 10.1021/es500690a

[mbt213280-bib-0081] Speers, A. M. , Schindler, B. D. , Hwang, J. , Genc, A. , and Reguera, G. (2016) Genetic identification of a PilT motor in *Geobacter sulfurreducens* reveals a role for pilus retraction in extracellular electron transfer. Front Microbiol 7: 1578. 27799920 10.3389/fmicb.2016.01578 PMC5065972

[mbt213280-bib-0082] Steidl, R. , Lampa‐Pastirk, S. , and Reguera, G. (2016) Mechanistic stratification in electroactive biofilms of *Geobacter sulfurreducens* mediated by pilus nanowires. Nature Comm 7: 12217. 10.1038/ncomms12217 PMC4974642 27481214

[mbt213280-bib-0083] Straub, K.L. , Kappler, A. and Schink, B. (2005) Enrichment and isolation of ferric‐iron‐ and humic‐acid‐reducing bacteria In Mehods in Enzymology. Leadbetter J.R. (ed.). Amsterdam, the Netherlands: Elsevier Academic Press, pp. 58–77. 10.1016/S0076-6879(05)97004-3 16260285

[mbt213280-bib-0084] Strycharz‐Glaven, S. M. , and Tender, L. M. (2012) Reply to the ‘Comment on “On electrical conductivity of microbial nanowires and biofilms”‘ by N. S. Malvankar, M. T. Tuominen and D. R. Lovley, Energy Environ. Sci., 2012, 5, doi: 10.1039/c2ee02613a . Energy Environ Sci 5: 6250–6255.

[mbt213280-bib-0085] Stubbe, J. (2003) Di‐iron‐tyrosyl radical ribonucleotide reductases. Curr Opin Chem Biol 7: 183–188. 12714050 10.1016/s1367-5931(03)00025-5

[mbt213280-bib-0086] Stubbe, J. , Nocera, D. G. , Yee, C. S. , and Chang, M. C. (2003) Radical initiation in the class I ribonucleotide reductase: long‐range proton‐coupled electron transfer? Chem Rev 103: 2167–2201. 12797828 10.1021/cr020421u

[mbt213280-bib-0087] Subramanian, P. , Pirbadian, S. , El‐Naggar, M. Y. , and Jensen, G. J. (2018) Ultrastructure of *Shewanella oneidensis* MR‐1 nanowires revealed by electron cryotomography. Proc Natl Acad Sci USA 115: E3246–E3255. 29555764 10.1073/pnas.1718810115 PMC5889646

[mbt213280-bib-0088] Sutter, M. , Greber, B. , Aussignargues, C. , and Kerfeld, C. A. (2017) Assembly principles and structure of a 6.5‐MDa bacterial microcompartment shell. Science 356: 1293–1297. 28642439 10.1126/science.aan3289 PMC5873307

[mbt213280-bib-0089] Tao, K. , Makam, P. , Aizen, R. and Gazit, E. (2017) Self‐assembling peptide semiconductors. Science 358, eaam9756. 29146781 10.1126/science.aam9756 PMC5712217

[mbt213280-bib-0090] Templin, M. F. , Stoll, D. , Schrenk, M. , Traub, P. C. , Vohringer, C. F. , and Joos, T. O. (2002) Protein microarray technology. Trends Biotechnol 20: 160–166. 11906748 10.1016/s0167-7799(01)01910-2

[mbt213280-bib-0091] Turro, N. J. , and Barton, J. K. (1998) Paradigms, supermolecules, electron transfer and chemistry at a distance. What's the problem? The science or the paradigm? J Biol Inorg Chem 3: 201–209.

[mbt213280-bib-0092] Uetrecht, C. , Barbu, I. M. , Shoemaker, G. K. , van Duijn, E. , and Heck, A. J. (2011) Interrogating viral capsid assembly with ion mobility‐mass spectrometry. Nat Chem 3: 126–132. 21258385 10.1038/nchem.947

[mbt213280-bib-0093] Vargas, M. , Malvankar, N. S. , Tremblay, P. L. , Leang, C. , Smith, J. A. , Patel, P. , *et al* (2013) Aromatic amino acids required for pili conductivity and long‐range extracellular electron transport in *Geobacter sulfurreducens* . MBio 4: e00105–e00113. 23481602 10.1128/mBio.00105-13 PMC3604773

[mbt213280-bib-0094] Veazey, J. P. , Reguera, G. , and Tessmer, S. H. (2011) Electronic properties of conductive pili of the metal‐reducing bacterium *Geobacter sulfurreducens* probed by scanning tunneling microscopy. Phys Rev E 84: 060901. 10.1103/PhysRevE.84.060901 22304032

[mbt213280-bib-0095] Winther‐Larsen, H. C. , Wolfgang, M. C. , van Putten, J. P. , Roos, N. , Aas, F. E. , Egge‐Jacobsen, W. M. , *et al* (2007) *Pseudomonas aeruginosa* Type IV pilus expression in *Neisseria gonorrhoeae*: effects of pilin subunit composition on function and organelle dynamics. J Bacteriol 189: 6676–6685. 17573479 10.1128/JB.00407-07 PMC2045180

[mbt213280-bib-0096] Xiao, K. , Malvankar, N. S. , Shu, C. J. , Martz, E. , Lovley, D. R. , and Sun, X. (2016) Low energy atomic models suggesting a pilus structure that could account for electrical conductivity of *Geobacter sulfurreducens* pili. Sci Rep 6: 23385. 27001169 10.1038/srep23385 PMC4802205

[mbt213280-bib-0097] Yamahata, C. , Collard, D. , Takekawa, T. , Kumemura, M. , Hashiguchi, G. , and Fujita, H. (2008) Humidity dependence of charge transport through DNA revealed by silicon‐based nanotweezers manipulation. Biophys J 94: 63–70. 17827222 10.1529/biophysj.107.115980 PMC2134877

[mbt213280-bib-0098] Yang, L. , Liu, A. , Cao, S. , Putri, R. M. , Jonkheijm, P. , and Cornelissen, J. J. L. M. (2016) Self‐assembly of proteins: Towards supramolecular materials. Chem Eur J 22: 15570–15582. 27535817 10.1002/chem.201601943

[mbt213280-bib-0099] Yates, M. D. , Golden, J. P. , Roy, J. , Strycharz‐Glaven, S. M. , Tsoi, S. , Erickson, J. S. , *et al* (2015) Thermally activated long range electron transport in living biofilms. Phys Chem Chem Phys 17: 32564–32570. 26611733 10.1039/c5cp05152e

[mbt213280-bib-0100] Yazici, H. , Fong, H. , Wilson, B. , Oren, E. E. , Amos, F. A. , Zhang, H. , *et al* (2013) Biological response on a titanium implant‐grade surface functionalized with modular peptides. Acta Biomater 9: 5341–5352. 23159566 10.1016/j.actbio.2012.11.004 PMC4410049

[mbt213280-bib-0101] Yee, C. S. , Chang, M. C. , Ge, J. , Nocera, D. G. , and Stubbe, J. (2003) 2,3‐difluorotyrosine at position 356 of ribonucleotide reductase R2: a probe of long‐range proton‐coupled electron transfer. J Am Chem Soc 125: 10506–10507. 12940718 10.1021/ja036242r

